# P-2168. The Intervention of Infectious Diseases Physicians Might Enhance the Value of Broad-Range 16S rRNA Gene Polymerase Chain Reaction in the Clinical Aspect

**DOI:** 10.1093/ofid/ofae631.2322

**Published:** 2025-01-29

**Authors:** Risa Fukuoka, Hidenori Nakagawa, Masaru Kato, Michinori Shirano

**Affiliations:** Osaka City General Hospital, Kobe, Hyogo, Japan; Osaka City General Hospital, Kobe, Hyogo, Japan; Osaka City General Hospital, Kobe, Hyogo, Japan; Osaka City General Hospital, Kobe, Hyogo, Japan

## Abstract

**Background:**

Evaluation of Broad-Range 16S rRNA gene polymerase chain reaction (BRPCR) has been reported to aim in diagnosing infection by detecting and identifying bacterial genes in clinical specimens from patients with suspected infections. However, collecting the right specimens at the right time and determining whether the bacteria detected by BRPCR are the true cause or contamination requires infectious disease expertise. We investigate whether there is an association between the detection of bacteria that caused infection using BRPCR and intervention by Infectious Diseases (ID) physicians.

**Methods:**

We reviewed the patients who underwent BRPCR at Osaka City General Hospital between April 2017 and March 2024. The collected items are age, sex, bacteria detected by BRPCR, determination of whether the bacteria if detected, caused infection, or were contaminated, and with or without the intervention of ID physicians (consultation to ID physicians). Then we analyzed the correlation between the intervention of ID physicians and the identification of the bacteria that caused the infection by BRPCR using Fisher's exact test. We also investigated whether the BRPCR results had affected the final diagnosis.

**Results:**

There was a total of 149 clinical specimens from 138 patients. Of these, 84 (60%) were male, with a median age of 48 years (IQR 6-72). Bacteria were identified in 101 (67.7%) clinical specimens. In 70 specimens of them, the identified bacteria were presumed to have caused the infection. Of the 70 specimens, 56 had interventions by ID physicians. Whereas there were 79 specimens in which bacteria were identified but were presumed to be contaminants, and in which bacteria were not identified, of which 49 had intervention by ID physicians. The results showed an association between the intervention of ID physicians and the identification of the bacteria that caused the infection by BRPCR (p=0.0196). In 29 (19.4%), the final diagnosis changed from infectious to non-infectious based on the results of BRPCR.

**Conclusion:**

Interventions by ID physicians might relate to identifying the bacteria causing an infection using BRPCR, and that might lead to enhancing the value of BRPCR.

**Disclosures:**

All Authors: No reported disclosures

